# The efficacy and safety of anti-EGFR target agents in patients with potentially resectable metastatic colorectal cancer: a meta-analysis of randomized controlled trials

**DOI:** 10.1186/s12957-023-03222-3

**Published:** 2023-10-26

**Authors:** Yang Wang, Xiangyuan Li, Tongmin Huang, Dongying Wang, Yujing He, Mengfei Wei, Yujie Chen, Matao Zheng, Yetan Shi, Jianjian Zhang

**Affiliations:** 1 Department of Pathology, Linhai Second People’s Hospital, Taizhou, Zhejiang China; 2 Department of Colorectal Surgery and Oncology, Key Laboratory of Cancer Prevention and Intervention, Ministry of Education, The Second Affiliated Hospital, Zhejiang University School of Medicine, Hangzhou, Zhejiang China; 3 The Second Clinical Medical College, Zhejiang Chinese Medical University, Hangzhou, Zhejiang China; 4 The First Clinical Medical College, Zhejiang Chinese Medical University, Hangzhou, Zhejiang China; 5 Department of Gastroenterology, Linhai Second People’s Hospital, 198 Dubei Road, Linhai, Taizhou, 317016 Zhejiang China

**Keywords:** Anti-epidermal growth factor receptor (anti-EGFR) target agent, Cetuximab; Panitumumab, Conversion therapy, Colorectal cancer, Colorectal cancer metastases

## Abstract

**Background:**

Adding anti-epidermal growth factor receptor (anti-EGFR) target agents to conversion therapy may improve the resection rates and survival of patients with potentially resectable metastatic colorectal cancer (mCRC). This study aims to analyze the efficacy and safety of additional anti-EGFR target agents.

**Methods:**

A systematic search was conducted on PubMed, Web of Science, Embase, and Cochrane Library. And all relevant studies published in English before January 2023 were collected to explore the impact of additional anti-EGFR targeted agent on the efficacy and safety of patients with potentially resectable mCRC (PROSPERO: CRD42022340523, https://www.crd.york.ac.uk/PROSPERO/).

**Results:**

This study included a total of 8 articles, including 2618 patients. The overall response rate (ORR) and R0 resection rates of the experimental group were higher than those of the control group, while there was no significant difference in progression-free survival (PFS) and overall survival (OS) between the two groups. In RAS/KRAS wild-type patients, the ORR (*RR*: 1.20, 95% *Cl*: 1.02–1.41, *p* = 0.03), R0 resection rate (*RR*: 1.60, 95% *Cl*: 1.17–2.20, *p* = 0.003), PFS (*HR*: 0.80, 95% *Cl*: 0.68–0.93, *p* = 0.003), and OS (*HR*: 0.87, 95% *Cl*: 0.76–0.99, *p* = 0.031) of the experimental group were higher than those of the control group. While in KRAS mutant patients, there was no statistical difference between the two groups in ORR, R0 resection rate, PFS, and OS.

**Conclusion:**

The addition of anti-EGFR targeted agents can improve the prognosis of RAS/KRAS wild-type patients with potentially resectable mCRC, while KRAS mutant patients may not benefit. In addition, the overall safety factor was controllable.

**Supplementary Information:**

The online version contains supplementary material available at 10.1186/s12957-023-03222-3.

## Introduction

As the third most common tumor in the world, colorectal cancer (CRC) was diagnosed in approximately 20 billion cases in 2020 and kill nearly 10 billion people each year, ranking it second in cancer-related mortality (https://www.iarc.fr/faq/latest global-cancer-data-2020-qa/). About 33% of patients with CRC will have metastases, with the most common metastatic site being liver metastasis, significantly reducing the expected 5-year overall survival rate (OS) and becoming the main cause of death [1, 2]. For patients with metastatic colorectal cancer (mCRC), complete surgical resection of the metastatic site is the only possible treatment, allowing the patient to achieve an almost radical effect. However, some patients have tumors that were initially unresectable due to the location, number, size, or other influencing factors of their tumors [3]. In recent years, the advances in chemotherapy and targeted drugs have opened up the possibility of conversion therapy for mCRC. Specifically, conversion therapy is systematic treatment administered preoperatively to reduce the size of the tumor and convert the initially unresectable metastatic lesions into resectable ones [4]. Moreover, the prognosis of patients who underwent resection after transformation was almost the same as that of patients who underwent initial resection [5, 6].

Conversion therapy is performed by chemotherapy with or without targeted drugs. The specific treatment plan needs to be formulated according to the status of patients, the mutation type of the tumor, and the location of the primary tumor (left side or right side) [7, 8]. In addition, for some giant liver metastases, some studies have suggested that hepatic artery intubation chemotherapy and hepatic artery embolization chemotherapy can also achieve high response rates and R0 resection rates [9]. Recently, studies have shown improved R0 resection rates as well as survival in patients treated with targeted therapy in combination with chemotherapy [10, 11], but the randomized phase 3 COIN study did not report that the addition of targeted therapeutic agents to the treatment regimen increased R0 resection rates [12]. In addition, different molecular subtypes respond differently to drugs [13]. Anti-epidermal growth factor receptor (anti-EGFR) target agent plus cytotoxic drugs are one of the conversion therapy for potentially resectable mCRC. They are monoclonal antibodies, including cetuximab and panitumumab, which target the EGFR receptor and block intracellular signaling, thereby inhibiting cancer cell proliferation [14].

There are few meta-analyses analyzing the role of anti-EGFR targeted agents in patients with potentially resectable mCRC, and although both cetuximab and panitumumab are anti-EGFR targeted agents, there are limited data assessing the efficacy and safety of panitumumab in patients with potentially resectable mCRC. Therefore, the present study focused on the efficacy and safety of adding anti-EGFR targeted agents in patient with potentially resectable mCRC.

## Methods

### Search strategy

Four electronic databases, including PubMed, Embase, Cochrane, and Web of Science, have been researched. This systematic study explored the efficacy and safety of anti-EGFR targeted agents in combination with chemotherapy in patients with potentially resectable mCRC. Articles published in English from inception until January 2023 were searched. The following search terms and keywords were used in the retrieval process: “anti-EGFR targeted agents,” “epidermal growth factor receptor targeted agents,” “panitumumab,” “cetuximab,” “colorectal liver metastasis,” and “metastatic colorectal cancer” (Supplementary Table 1). In addition, data could also be derived from the reference lists of studies, and this meta-analysis complies with the Preferred Reporting Items Systematic reviews and Meta-Analyses (PRISMA) guidelines for the systematic review and meta-analysis [15]. The study protocol has registered with the PROSPERO: CRD42022340523 (https://www.crd.york.ac.uk/PROSPERO/).

### Selection criteria

Inclusion criteria were based on PICOS principles (participants, intervention, comparison, outcomes, and study design): (1) participants: patients with potentially resectable mCRC; (2) intervention: use of anti-EGFR targeted agents in combination with chemotherapy in the experimental group; (3) comparison: patients in the control group received chemotherapy only; (4) outcomes: data on at least one of the progression-free survival (PFS), overall survival, and objective response rate (ORR) was reported in results; and (5) study design: randomized controlled trial (RCT).

The following exclusion criteria were used: (1) patients with resectable mCRC were included in the study; (2) R0 resection rate was not reported in study; (3) data could not be extracted, or the extracted data could not be further processed; and (4) the article was not published in English. If the studies were conducted for the same clinical trial, the present study would contain the most recent or comprehensive version.

In general, the present study requires that included in the study must be patients with mCRC who are likely to be resected after conversion therapy and excludes patients with initially resectable mCRC.

### Data extraction and quality assessment

Articles that met the inclusion criteria were further analyzed by two independent authors. Extracted information was recorded in a standardized Microsoft Excel form (Microsoft Corp): author, year of publication, country, number of patients, treatment regimen, follow-up time, and prognostic outcomes of the studies. Any discrepancy between the two independent authors was judged by a third researcher to reach consensus. The meta-analysis concentrates on prognostic endpoints, including PFS, OS, ORR, R0 resection, and adverse events (AEs). In addition, the quality of RCTs was evaluated by Cochrane Collaboration’s tool in seven aspects: random sequence generation, allocation concealment, blinding of participants and personnel, blinding of outcome assessment, incomplete outcome data, selective reporting, and other bias. The risk of bias was classified as “high risk,” “low risk,” and “unclear risk.” In the end, only high-quality articles were included in the analysis.

### Statistical analysis

To statistically analyze the prognosis and surgical resection rate of anti-EGFR targeted agents combined with chemotherapy, hazard ratio (HR) as well as 95% confidence intervals (CI) for PFS and OS was extracted from the included articles in this study to evaluate the prognostic impact of anti-EGFR targeted agents plus chemotherapy. Furthermore, ORR, R0 resection, and AEs were measured by risk ratio (RR) and 95%CI. Statistical heterogeneity was determined by using chi-square test and *I*^2^ statistic, with *p* < 0.1 or *I*^2^ > 50% indicating high heterogeneity. Due to the different conditions of patients and the use of different drugs, the random-effect model was used to improve the reliability of the results. Begg’s test was used to evaluate publication bias. Sensitivity analysis was used to verify the stability of the results. All the statistical tests were performed with RevMan 5.4 and STATA version 10.0 (Stata Corporation, College Station, TX, USA). The *p*-value is two sided, and the results of *p* < 0.05 were deemed to be statistically significant.

## Results

### Eligible research and inclusion characteristics

**Fig. 1:**
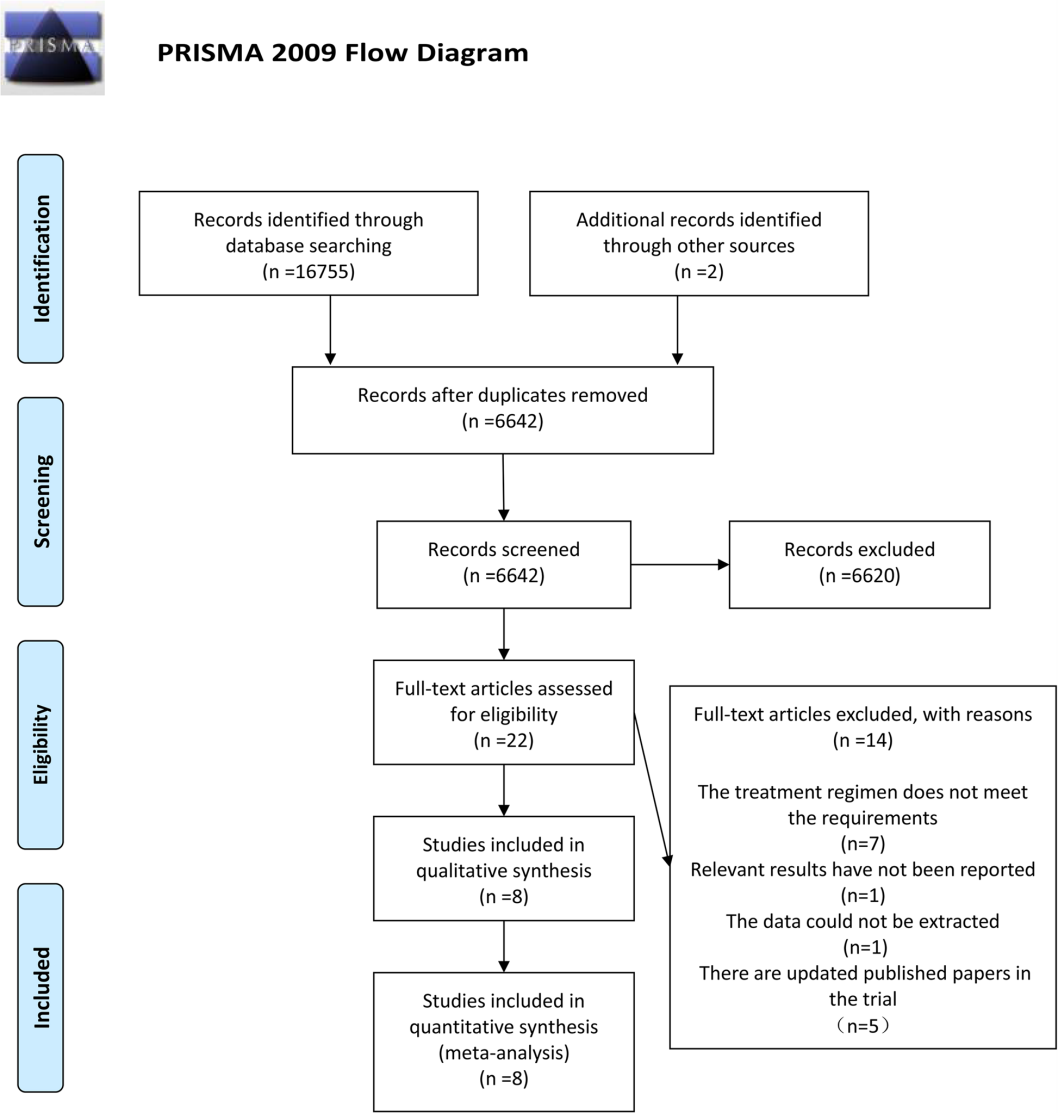
Flow diagram describing inclusion and exclusion criteria

Figure 1 shows the flow chart of the screening process. A total of 16,757 citations were obtained by searching PubMed, Cochrane, Embase, and Web of Science as well as two articles obtained from the references of other articles. After the removal of duplicate citations, 6642 citations remained. Among them, 6620 citations were excluded because their titles or abstracts obviously did not meet the inclusion criteria of present study. After reading the full text of the remaining 22 articles, we found that 14 of the 22 articles were excluded due to the following reasons: 7 articles used treatment regimens that did not meet the requirements, 5 studies were excluded due to the discovery of updated publication on the same trail, 1 article did not report the relevant results required for this study, and the data of 1 article could not be extracted. Therefore, 8 articles [10–13, 16–19] with a total of 2618 patients were finally included in this meta-analysis.

The 8 studies included in present study were RCTs, including 6 studies from Europe and 2 studies from Asia. All patients in the experimental group received anti-EGFR targeted agents in combination with chemotherapy, with cetuximab in 6 studies and panitumumab in the other 2 studies. As far as the chemotherapy regime was concerned, only 1 study of the chemotherapy regime used triplet chemotherapy regimen, and the other 7 studies used doublet chemotherapy. All 8 articles reported the PFS, OS, and ORR of patients after treatment. More detailed information is provided in Table 1 and Supplementary Table 2. Detailed information on the quality assessment of the 8 articles included in present study is shown in Supplementary Fig. 1 and Supplementary Fig. 2.

**Table 1:** Characteristics of all the studies included in the meta-analysis

Author	Year	Country	Treatment regimens		Number of patients		Follow-up (months)	Outcomes
			Experiment	Control	Experiment	Control		
Van Cutsem	2009	Belgium	Cetuximab + FOLFIRI	FOLFIRI	599	599	Experiment: 29.9 Control: 29.4	PFS OS ORR R0 resection rate
Bokemeyer	2011	Germany	Cetuximab + FOLFOX-4	FOLFOX-4	169	168	NA	PFS OS ORR R0 resection rate
Maughan	2011	England	Oxaliplatin and fluoropyrimidine + cetuximab	Oxaliplatin and fluoropyrimidine	815	815	Experiment: 78 Control: 76	PFS OS ORR R0 resection rate
Tveit	2012	Norway	Cetuximab + Nordic FLOX	Nordic FLOX	194	185	NA	PFS OS ORR R0 resection rate
Ye	2013	China	Cetuximab + FOLFIRI or mFOLFOX6	FOLFIRI or mFOLFOX6	70	68	25.0	PFS OS ORR R0 resection rate
Qin	2018	China	Cetuximab + FOLFOX-4	FOLFOX-4	193	200	Experiment: 44.4 Control: 48.7	PFS OS ORR R0 resection rate
Modest	2019	Germany	Panitumumab + mFOLFOXIRI	mFOLFOXIRI	63	33	Experiment: 44.2 Control: 66.3	PFS OS ORR R0 resection rate
Douillard	2014	France	Panitumumab + FOLFOX4	FOLFOX4	546	550	Experiment: 15.3 Control: 18.5	PFS OS ORR R0 resection rate

*FOLFIRI* Irinotecan, fluorouracil, and leucovorin, *FOLFOX-4* Leucovorin, fluorouracil, and oxaliplatin, *FOLFOXIRI* Fluorouracil/folinic acid, oxaliplatin, and irinotecan, *FLOX* Bolus fluorouracil/folinic acid and oxaliplatin, *FOLFOX* 5-fluorouracil, folinic acid, and oxaliplatin, *NA* Not available, *PFS* Progression-free survival, *OS* Overall survival, *ORR* Objective response rate

### Objective response rate and R0 resection rate

Eight articles reported the ORR and R0 resection rates of patients. In general, patients receiving anti-EGFR targeted agents combined with chemotherapy have higher ORR (*RR*: 1.24, 95% *Cl*: 1.13–1.37, *p* < 0.001) and R0 resection rate (*RR*: 1.63, 95% *Cl*: 1.27–2.09, *p* < 0.001) than those in the control group. Among RAS/KRAS wild-type patients, the ORR (*RR*: 1.20, 95% *Cl*: 1.02–1.41, *p* = 0.03) and R0 resection rate (*RR*: 1.60, 95% *Cl*: 1.17–2.20, *p* = 0.003) were higher in the experimental group than in the control group. In this study, 4 articles provided ORR after treatment in patients with KRAS mutant, and 2 articles provided R0 resection rate. Among patients with KRAS mutant, there was no statistically significant difference in ORR (*RR*: 0.99, 95% *Cl*: 0.78–1.25, *p* = 0.94) and R0 resection rate (*RR*: 2.90, 95% *Cl*: 0.46–18.54, *p* = 0.26) between the experimental group and the control group. Details are shown in Table 2.

**Table 2:** Subgroup analysis of ORR and R0 resection

Outcomes	No. of studies	*RR*	95% *CI*	*P*	Heterogeneity	
					*I*^2^	*P*
ORR	8	1.24	1.13, 1.37	< 0.001	52%	0.03
RAS/KRAS wild type	8	1.20	1.02, 1.41	0.03	81%	< 0.001
KRAS mutant	4	0.99	0.78, 1.25	0.94	64%	0.04
Cetuximab	6	1.28	1.13, 1.45	< 0.001	58%	0.04
Panitumumab	2	1.18	0.98, 1.42	0.08	55%	0.11
R0 resection	8	1.63	1.27, 2.09	< 0.001	0	0.48
RAS/KRAS wild type	8	1.60	1.17, 2.20	0.003	23%	0.25
KRAS mutant	2	2.90	0.46, 18.54	0.26	18%	0.27
Cetuximab	6	1.69	1.26, 2.28	< 0.001	0	0.50
Panitumumab	2	1.66	0.80, 3.43	0.18	48%	0.17

R0 resection, complete resection; *ORR* Overall response rate, *RR* Risk ratio, *CI* Confidence interval

In addition, according to the drug types of anti-EGFR targeted agents, data on ORR and R0 resection rates in patients treated with cetuximab combined with chemotherapy were mentioned in 6 articles, and the ORR and R0 resection rates of patients treated with panitumumab combined with chemotherapy were mentioned in 2 articles. Compared to the control group, statistical analysis showed that patients receiving cetuximab in combination with chemotherapy had higher ORR (*RR*: 1.28, 95% *Cl*: 1.13–1.45, *p* < 0.001) and R0 resection rate (*RR*: 1.69, 95% *Cl*: 1.26–2.28, *p* < 0.001). There was no significant difference in ORR (*RR*: 1.18, 95% *Cl*: 0.98–1.42, *p* = 0.08) and R0 resection rate (*RR*: 1.66, 95% *Cl*: 0.80–3.43, *p* = 0.18) between patients in the experimental group receiving panitumumab in combination with chemotherapy and those in the control group receiving chemotherapy only. Details are shown in Table 2.

### Effect of anti-EGFR targeted agents on survival

**Fig. 2:**
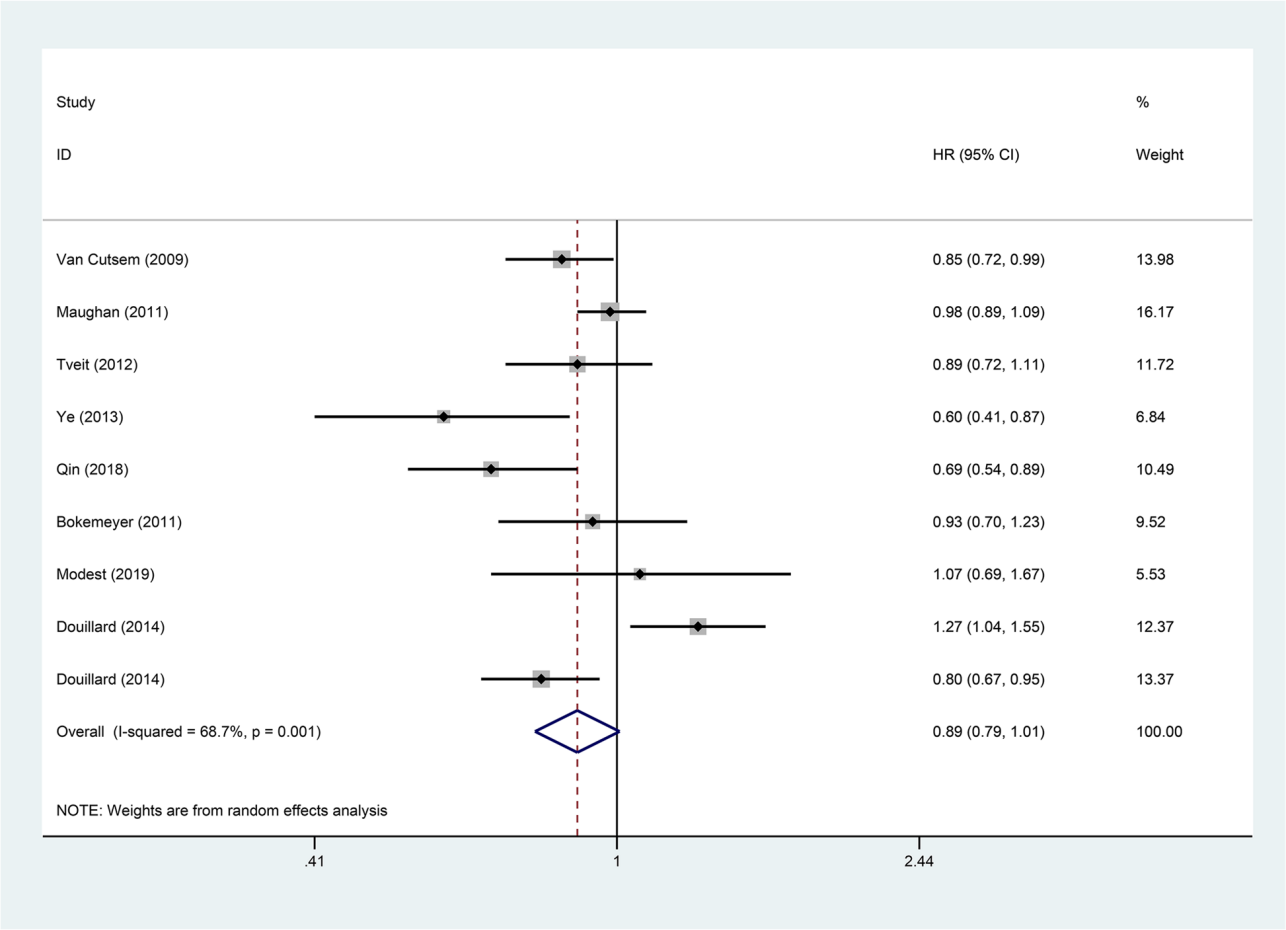
Forest plots of the PFS for additional anti-EGFR target agents on mCRC (*p* = 0.066)

**Fig. 3:**
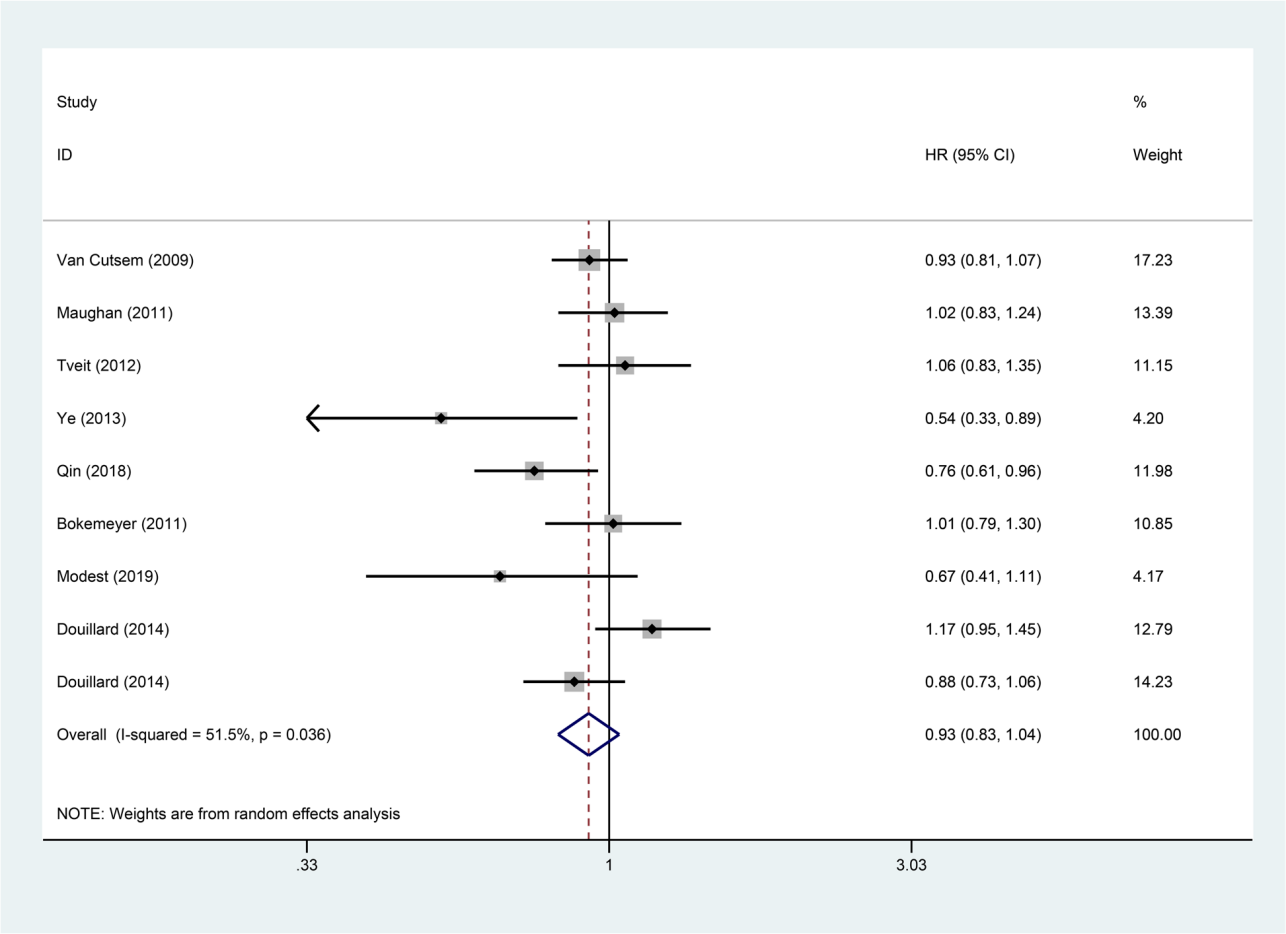
Forest plots of the OS for additional anti-EGFR target agents on mCRC (*p* = 0.188)

**Fig. 4:**
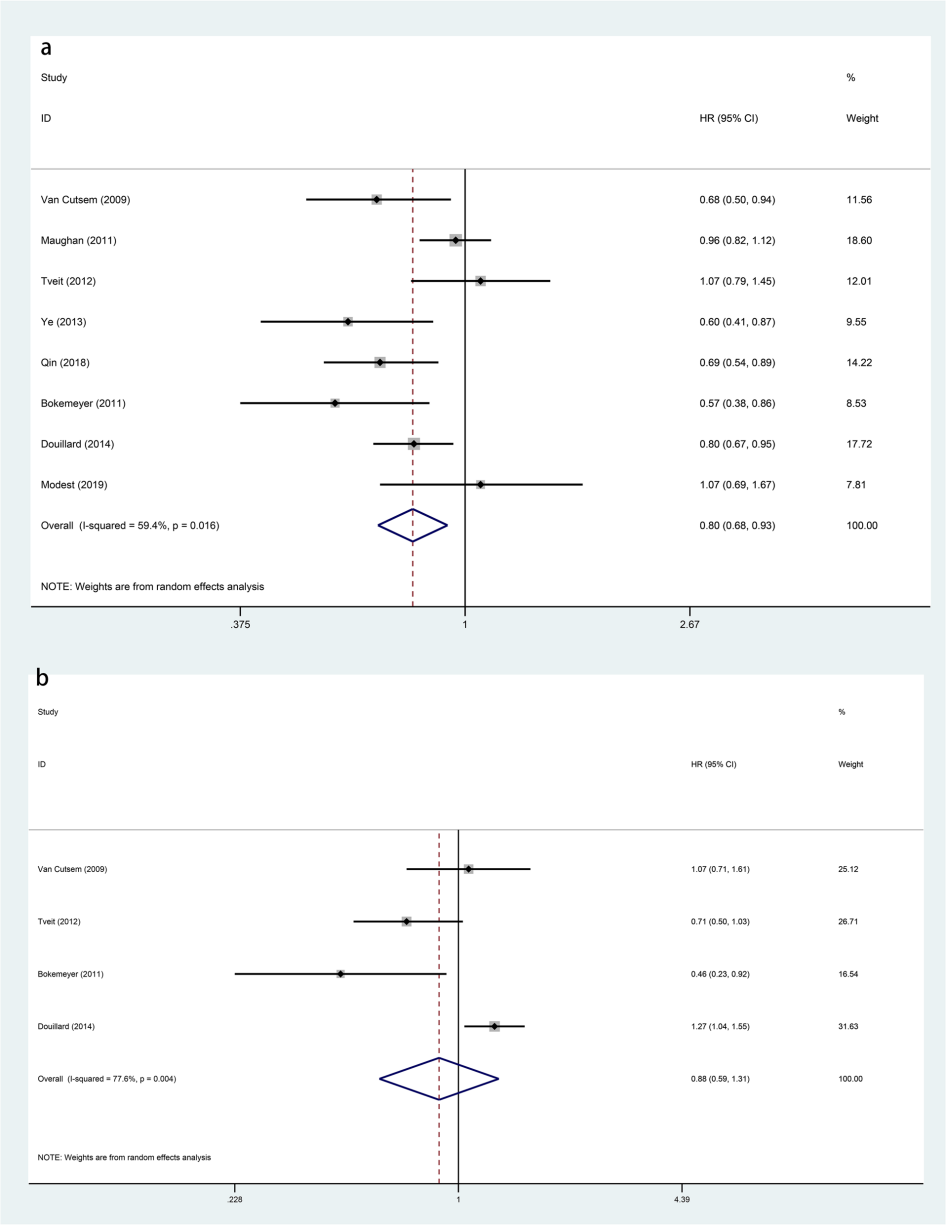
Forest plots of subgroup analysis of the PFS for additional anti-EGFR target agents on mCRC **a** RAS/KRAS wild-type patients (*p* = 0.003). **b** KRAS mutant patients (*p* = 0.526)

**Fig. 5:**
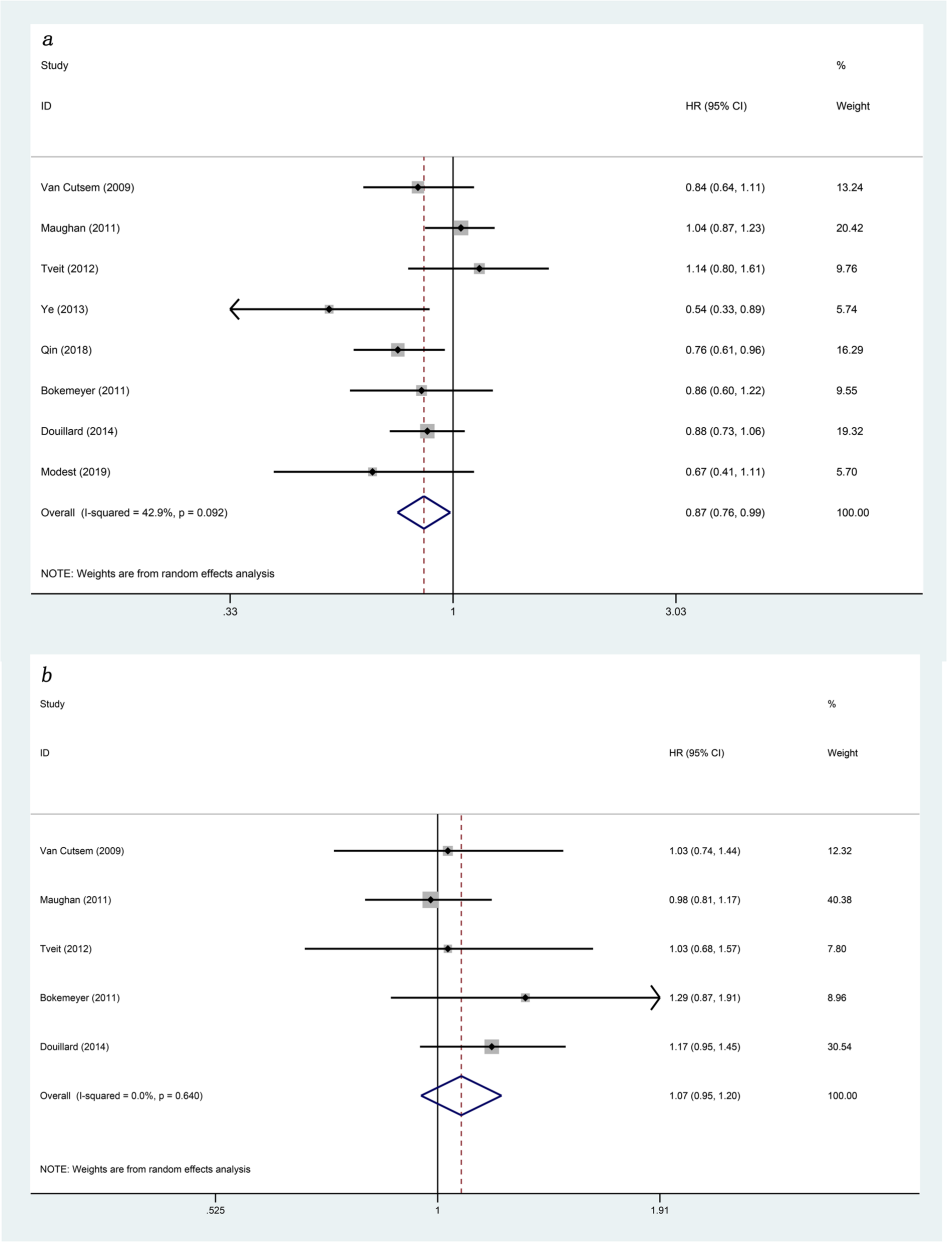
Forest plots of subgroup analysis of the OS for additional anti-EGFR target agents on mCRC **a** RAS/KRAS wild-type patients (*p* = 0.031). **b** KRAS mutant patients (*p* = 0.250)

The 8 articles included in present study provided detailed data on PFS and OS (Figs. 2 and  3). As for the PFS (*HR*: 0.89, 95% *Cl*: 0.79–1.01, *p* = 0.066) and OS (*HR*: 0.93, 95% *Cl*: 0.83–1.04, *p* = 0.188) of patients in the experimental group and the control group, the differences between the two were not statistically significant. In order to further explore the impact of genetic status on the prognosis of patients, this study divided them into RAS/KRAS wild type and KRAS mutant. In present study, 8 articles reported PFS of patients with RAS/KRAS wild type after treatment, and 4 articles reported PFS of patients with KRAS mutant. Statistical analysis showed that, comparing with the control group, the PFS was higher in patients with RAS/KRAS wild type in the experimental group (*HR*: 0.80, 95% *Cl*: 0.68–0.93, *p* = 0.003). However, for patients with KRAS mutant, there was no statistical difference in PFS between the two groups (*HR*: 0.88, 95% *Cl*: 0.59–1.31, *p* = 0.526). The OS for RAS/KRAS wild-type patients can be found in the 8 articles and for KRAS mutant patients in 5 articles. For RAS/KRAS wild-type patients, the OS was higher in the experimental group with anti-EGFR targeted agents than in the control group (*HR*: 0.87, 95% *Cl*: 0.76–0.99, *p* = 0.031). However, in patients with KRAS mutant, the difference between the two groups was not statistically significant (*HR*: 1.07, 95% *Cl*: 0.95–1.20, *p* = 0.250). More detailed information is in Figs. 4 and  5.

### Grade 3 or higher adverse events

In this study, 8 articles reported on relevant contents of AEs. Grade 3 or higher AEs are listed in Table 3. Four articles reported the total AEs of patients. The pooled analysis results showed that the incidence of AEs was higher in the experimental group than in the control group (*RR*: 1.22, 95% *Cl*: 1.05–1.42, *p* = 0.01). Compared with the control group, statistical analysis showed that anti-EGFR targeted agents with chemotherapy increased the incidence of some AEs in patients, such as diarrhea (*RR*: 1.71), fatigue (*RR*: 1.54), rash (*RR*: 26.60), skin reactions (*RR*: 44.52), hemoglobin (*RR*: 2.43), hypermagnesemia (*RR*: 13.10), stomatitis (*RR*: 2.90), cardiac events (*RR*: 29.62), and infusion-related reactions (*RR*: 2.32).

**Table 3:** Subgroup analysis of grade 3 or higher adverse events

	No. of studies	RR	95% *CI*	P	Heterogeneity	
					(*I*^2^)	*P*
Adverse events	4	1.22	1.05–1.42	0.01	63%	0.04
Neutropenia	7	1.03	0.89–1.20	0.65	65%	0.009
Febrile neutropenia	4	1.30	0.89–1.90	0.18	0	0.84
Leukopenia	6	1.14	0.81–1.61	0.46	57%	0.04
Diarrhea	8	1.71	1.49–1.97	< 0.001	0	0.78
Fatigue	6	1.54	1.20–1.99	< 0.001	0	0.49
Rash	5	26.60	4.93–143.62	< 0.001	81%	< 0.001
Skin reactions	4	44.52	18.49–107.20	< 0.001	24%	0.27
Vomiting	4	1.38	0.81–1.37	0.23	43%	0.15
Thrombocytopenia	4	1.18	0.80–1.76	0.41	0	0.48
Hemoglobin	4	2.43	1.44–4.11	< 0.001	0	0.68
Neurologic toxicities	4	0.82	0.63–1.08	0.16	36%	0.19
Hypomagnesemia	4	13.10	5.28–32.49	< 0.001	0	0.40
Stomatitis	4	2.90	1.76–4.78	< 0.001	0	0.43
Cardiac events	4	29.62	2.87–305.77	0.004	73%	0.01
Infusion-related reaction	5	2.32	1.04–5.17	0.04	31%	0.22

*RR* Risk ratio, *CI* Confidence interval

### Publication bias and sensitivity analysis

The present study evaluated the potential publication bias of articles reporting PFS and OS according to the genetic profile (RAS/KRAS wild type and RAS/KRAS mutant), and no significant publication bias was found. Details are shown in Supplementary Fig. 3 (*p* = 0.174), Supplementary Fig. 4 (*p* = 0.308), Supplementary Fig. 5 (*p* = 0.174), and Supplementary Fig. 6 (*p* = 0.806). In addition, the sensitivity analysis results of present study were generally stable. Details are shown in Supplementary Fig. 7, Supplementary Fig. 8, Supplementary Fig. 9, and Supplementary Fig. 10.

## Discussion

For mCRC patients with different initial conditions (initially resectable, potentially resectable, and unresectable), the addition of anti-EGFR targeted agents has different status. In turn, the determination of resectability needs to be evaluated by a professional multidisciplinary team [20, 21]. With the continuous progress of research, the applicability of resectable metastatic lesions is becoming more extensive [22]. At present, these definitions of resectability are only at the technical level and are based on the possibility of completely removing all visible metastatic tumors, leaving an adequately functioning parenchyma [23]. It includes the following: in terms of tumor, the tumor is required to be resectable, and the margin of resection is negative, and the tumor is required to respond to preoperative chemotherapy, and extrahepatic diseases should be controlled; in terms of liver condition, patients are required to have two contiguous functional liver segments with preserved blood flow of vein, artery, portal vein, and biliary tract; in addition, the number of liver lesions and other factors that are related to poor prognosis after conversion resection also needs attention [24–27].

For patients with initially resectable mCRC, in the New EPOC study, Bridgewater et al. found that patients receiving cetuximab in combination with chemotherapy had lower survival rates than those receiving chemotherapy alone. Therefore, this study suggests that cetuximab should not be used as neoadjuvant therapy for patients with resectable mCRC [28]. For patients with initially unresectable mCRC, there is currently no clear standard to distinguish between those who are suitable for palliative care only and those who have the opportunity to undergo surgery after receiving conversion treatment. Therefore, they may both be potentially resectable mCRC patients. However, with the progress of research, some patients with tumors (that were) previously considered unresectable can now benefit from the removal of metastatic lesions through conversion treatment. For example, in the VOLFI trial included in present study, some patients who were initially assigned to cohort 1 (where tumor resection was considered impossible) still achieved resection after receiving anti-EGFR targeted agents in combination with chemotherapy [13]. Moreover, even if the patient has a wider range of metastatic lesions beyond the liver, there is still a chance of achieving R0 resection after receiving conversion treatment [29]. This meta-analysis examines the impact of additional anti-EGFR targeted agents on the rate of conversion resection in patients with potentially resectable mCRC and analyzed the efficacy and safety of this treatment regime.

The pooled analysis results of present study showed that additional anti-EGFR targeted agents could improve ORR and R0 resection rates in patients with potentially resectable mCRC, which is consistent with the results of many previous studies [10, 13, 19]. A higher ORR may imply a higher rate of conversion resection [30]. From a genetic perspective, the addition of anti-EGFR targeted agents might improve the ORR and R0 resection rates of RAS/KRAS wild-type patients, but KRAS mutant patients do not received this benefit. Here, attention needs to be paid to the correlation between targeted therapy and genes. Innocenti et al. noted that low tumor mutation burden and RAS and BRAF mutations were negative prognostic factors in the first-line treatment for patients with mCRC [31]. Mutations in KRAS resulted in sustained activation of the protein it encoded, so that even if upstream EGFR overexpression is blocked, downstream events cannot be regulated, and tumor growth and proliferation continue [32]. And studies have shown that during the treatment of anti-EGFR targeted agents, RAS mutant clones may exist in the initially RAS wild-type tumors, leading to resistance to anti-EGFR targeted agents [33]. The exception was tumors carrying KRAS^G13D^ mutation, where neurofibromin (NF1) ensures the normal expression of KRAS protein, while KRAS^G13D^ has weak interaction with NF1 and cannot competitively inhibit NF1. Therefore, even if KRAS^G13D^ is mutated, it can still rely on EGFR to exert its effect [32].

In addition, the present study also explored the effects of different anti-EGFR targeted agents (cetuximab vs panitumumab) on ORR and R0 resection rates. The results showed that the addition of cetuximab could benefit patients in terms of response rate and resection rate, and the results of many previous studies also support this conclusion [11, 34]. Unfortunately, similar conclusion was not observed in the pooled analysis results related to panitumumab. The research data related to panitumumab was limited, and the impact of genotype on the outcomes still needs to be considered here. In the two trials included in this study, the effect of treatment with additional panizumab on mCRC was investigated. Modert et al. studied patients with RAS wild-type mCRC [13], whereas in the PRIME trial included in present study, both KRAS wild-type and KRAS mutant patients were included in it [17, 35]. Therefore, this suggested that KRAS testing was essential for selecting suitable patients for anti-EGFR targeted therapy.

In terms of survival, the results of the pooled analysis showed that the addition of anti-EGFR targeted agents did not improve PFS and OS in patients with potentially resectable mCRC. Based on different gene types, the results of the subgroup analysis indicated that RAS/KRAS wild-type patients could achieve survival benefits after receiving anti-EGFR targeted agents combined with chemotherapy, while KRAS mutant patients could not improve their PFS or OS through additional anti-EGFR targeted agents. These results are supported by many previous studies [36–38]. When exploring the relationship between response rate, resection rate, and prognosis, present study found that in RAS/KRAS wild-type patients, additional anti-EGFR targeted agents improved not only the ORR and R0 resection rates of patients but also their survival rates, while KRAS mutant patients did not benefit in these aspects. This may further emphasize the importance of molecular screening in transformation therapy, while more profound screening such as BRAF also needs to be taken seriously [39, 40].

In addition, the combined chemotherapy regimen can also have an impact on the prognosis of patients, with some studies suggesting that triplet chemotherapy can improve responses rates and tumor resection rates and bring survival benefits compared to doublet chemotherapy [22, 41, 42]. Also, it has been suggested that this intensive therapy can increase the incidence of AEs [22, 43]. Recently, Ychou et al. published a comparison of the results of doublet chemotherapy or triplet chemotherapy combined with targeted therapy. The subjects of this study were generally in good condition and had a median age of 60 years. However, no significant benefit in terms of tumor resection rate and OS was observed in the triplet chemotherapy group in this study compared to the doublet chemotherapy group [44], which was consistent with the findings of Carrato et al. [45]. This makes us wonder whether the benefits of triple drug chemotherapy have been overestimated.

In terms of the safety of anti-EGFR targeted agents combined with chemotherapy, present study showed that compared to simple chemotherapy, additional anti-EGFR targeted agents increased the incidence of AEs above grade 3, mainly manifested in diarrhea, fatigue, rash, skin reactions, hemoglobin, hypermagnesemia, stomatitis, cardiac events, and infusion-related reactions. The overall safety factor was manageable. Studies have suggested that higher levels of skin toxicity are associated with improved ORR, and that skin toxicity can be considered as a surrogate indicator of the efficacy of cetuximab [10, 19, 46].

There are some published meta-analyses similar to this study, but we found that there are still many differences between these studies and present study. In terms of intervention, the study by Kong et al. did not explore the impact of panitumumab on patients with potentially resectable mCRC, and there are some differences in retrieval strategies and data processing between present study and those of Kong et al., so the results need further confirmation [47]. As far as the participants in the study are concerned, participants in Li et al., Wu et al., and Mastrantoni et al. were mCRC patients [48, 49] and did not limit participants to patients with potentially resectable mCRC. Present study limited the participants to patients with potentially resectable mCRC and conducted a systematic search of four major databases and rigorous data analysis, ultimately including only data from RCT for analysis so that more targeted research can be conducted on these patients.

There are some limitations to present study: firstly, due to a lack of data, other molecular features of the tumor [16, 34], chemotherapy regimen [7], and the location of the primary tumor [50] were not explored in present study, all of which may have influenced the results. Secondly, the follow-up times of the trials included in present study was not consistent, and we were unable to evaluate whether there was a correlation between various outcomes and time. Nevertheless, present study is the first to systematically explore the efficacy and safety of additional anti-EGFR targeted agents in patients with potentially resectable mCRC using a meta-analysis. The impact of genotype and drug on outcomes were further explored by subgroup analysis.

## Conclusion

The meta-analysis showed that additional anti-EGFR targeted agents could improve ORR, R0 resection rate, and survival rate in RAS/KRAS wild-type patients with potentially resectable mCRC, with a manageable overall safety margin. Meanwhile, the results of present study demonstrate the importance of molecular screening. With the progress of research, more patients may be offered radical treatment under conversion therapy in the future.

### Supplementary Information

**Additional file 1: Supplementary Fig. 1. **A review of authors’ judgements about each risk of bias item for each included study.

**Additional file 2: Supplementary Fig. 2. **A review of authors’ judgements about each risk of bias item presented as percentages across all included studies.

**Additional file 3:  Supplementary Fig. 3. **Begg’s test of the PFS for additional anti-EGFR target agents on RAS/KRAS wild-type patients (p=0.174).

**Additional file 4:  Supplementary Fig. 4. **Begg’s test of the PFS for additional anti-EGFR target agents on KRAS mutant patients (p=0.308).

**Additional file 5: Supplementary Fig. 5. **Begg’s test of the OS for additional anti-EGFR target agents on RAS/KRAS wild-type patients (p=0.174).

**Additional file 6: Supplementary Fig. 6. **Begg’s test of the OS for additional anti-EGFR target agents on KRAS mutant patients (p=0.806).

**Additional file 7: Supplementary Fig. 7. **Sensitivity analysis of the PFS for additional anti-EGFR target agents on RAS/KRAS wild-type patients.

**Additional file 8: Supplementary Fig. 8. **Sensitivity analysis of the PFS for additional anti-EGFR target agents on KRAS mutant patients.

**Additional file 9: Supplementary Fig. 9. **Sensitivity analysis of the OS for additional anti-EGFR target agents on RAS/KRAS wild-type patients.

**Additional file 10: Supplementary Fig. 10. **Sensitivity analysis of the OS for additional anti-EGFR target agents on KRAS mutant patients.

**Additional file 11: Supplementary Table 1. **Literature search strategy.

**Additional file 12: upplementary Table 2.** Characteristics of all the studies included in the meta-analysis.

## Data Availability

The original contributions presented in the study are included in the article/supplementary material, and further inquiries can be directed to the corresponding authors.

## Abbreviations

CRC: Colorectal cancer
mCRC: Metastatic colorectal cancer
Anti-EGFR: Anti-epidermal growth factor receptor
ORR: Overall response rate
PFS: Progression-free survival
OS: Overall survival
RCT: Randomized controlled trial
AEs: Adverse events
HR: Hazard ratio
CI: Confidence intervals
RR: Risk ratio
NF1: Neurofibromin
