# Supplementary figures and images for: The efficacy and safety of anti-EGFR target agents in patients with potentially resectable metastatic colorectal cancer: a meta-analysis of randomized controlled trials
Source: World J Surg Oncol. 2023 Oct 26;21:340. doi: 10.1186/s12957-023-03222-3 (PMC10601219; doi:10.1186/s12957-023-03222-3)

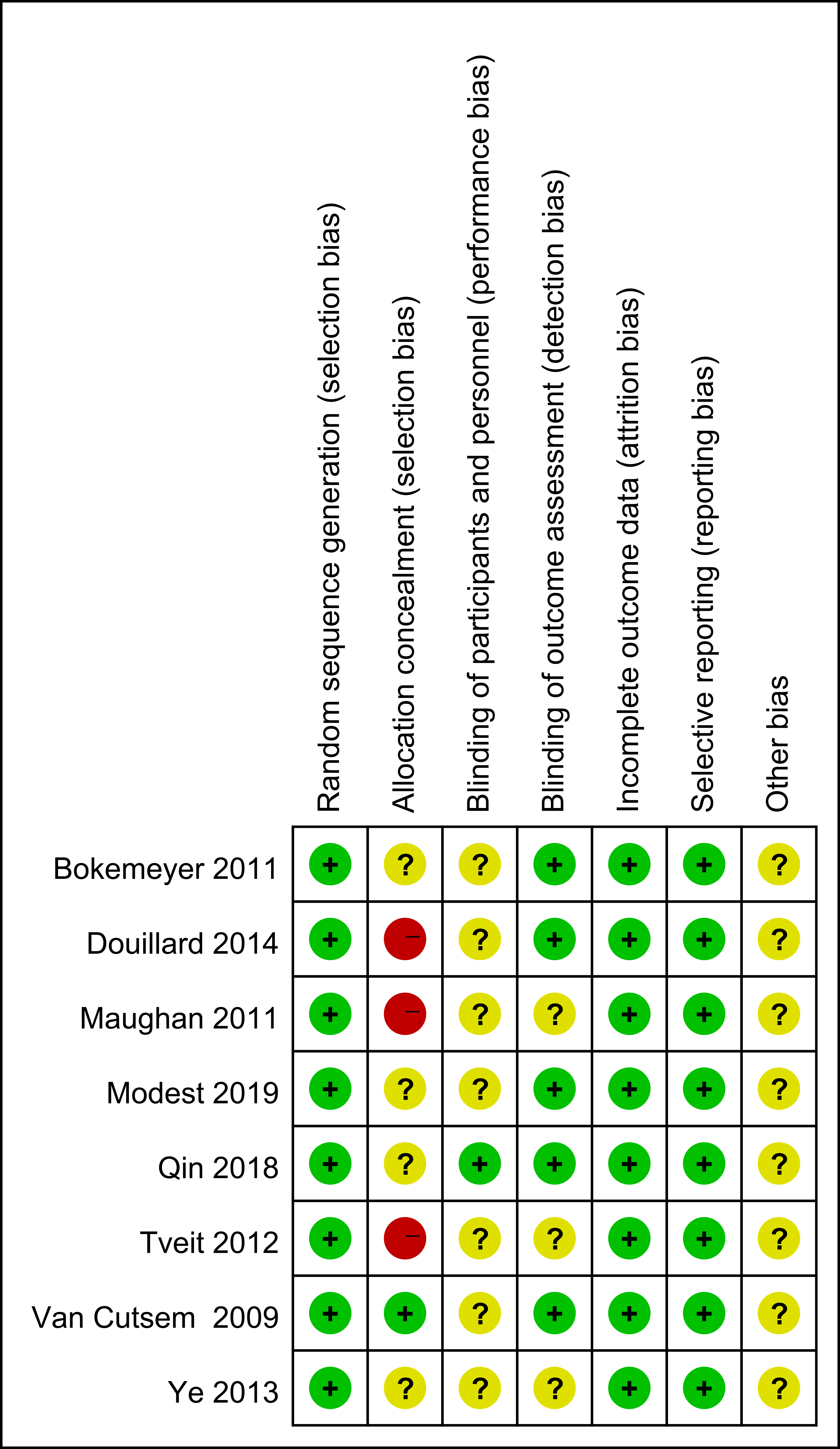

Supplement: Supplementary file 1 — Additional file 1: Supplementary Fig. 1. A review of authors’ judgements about each risk of bias item for each included study. [file 12957_2023_3222_MOESM1_ESM.tif]

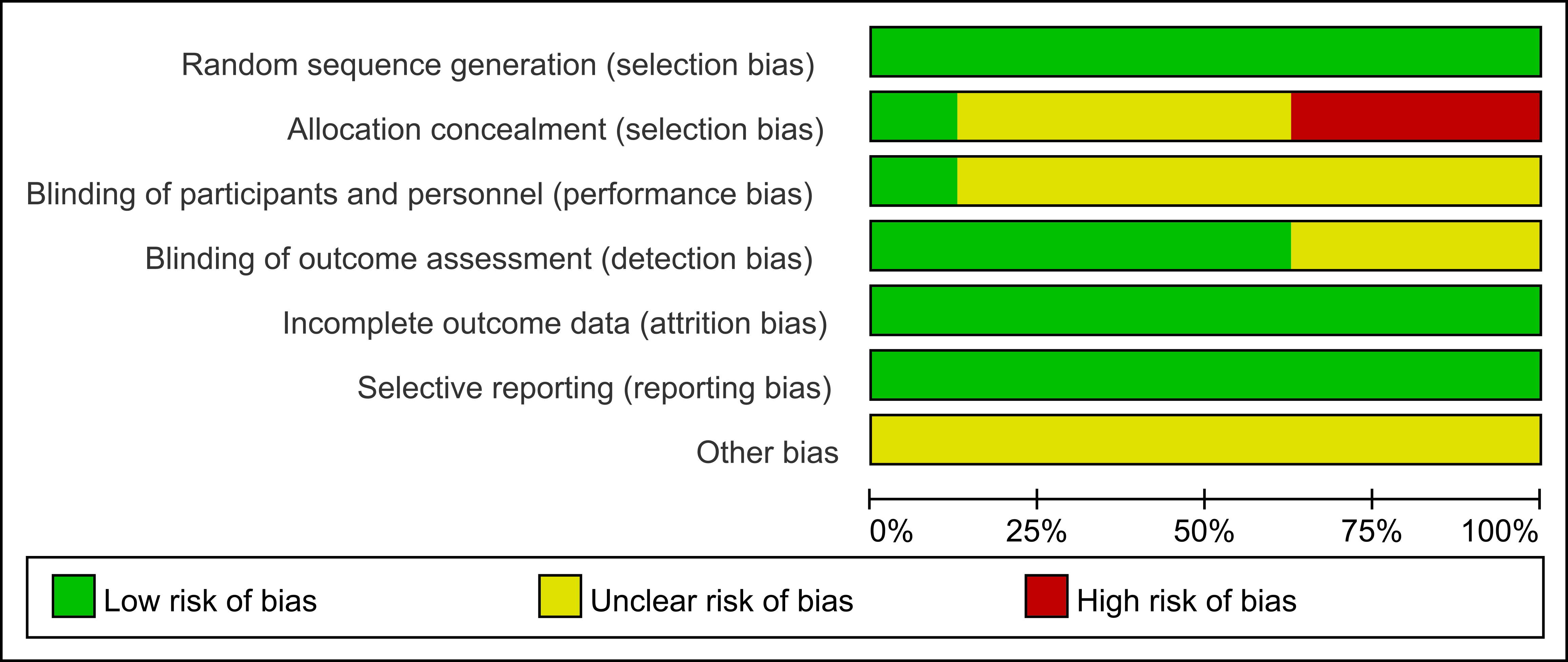

Supplement: Supplementary file 2 — Additional file 2: Supplementary Fig. 2. A review of authors’ judgements about each risk of bias item presented as percentages across all included studies. [file 12957_2023_3222_MOESM2_ESM.tif]

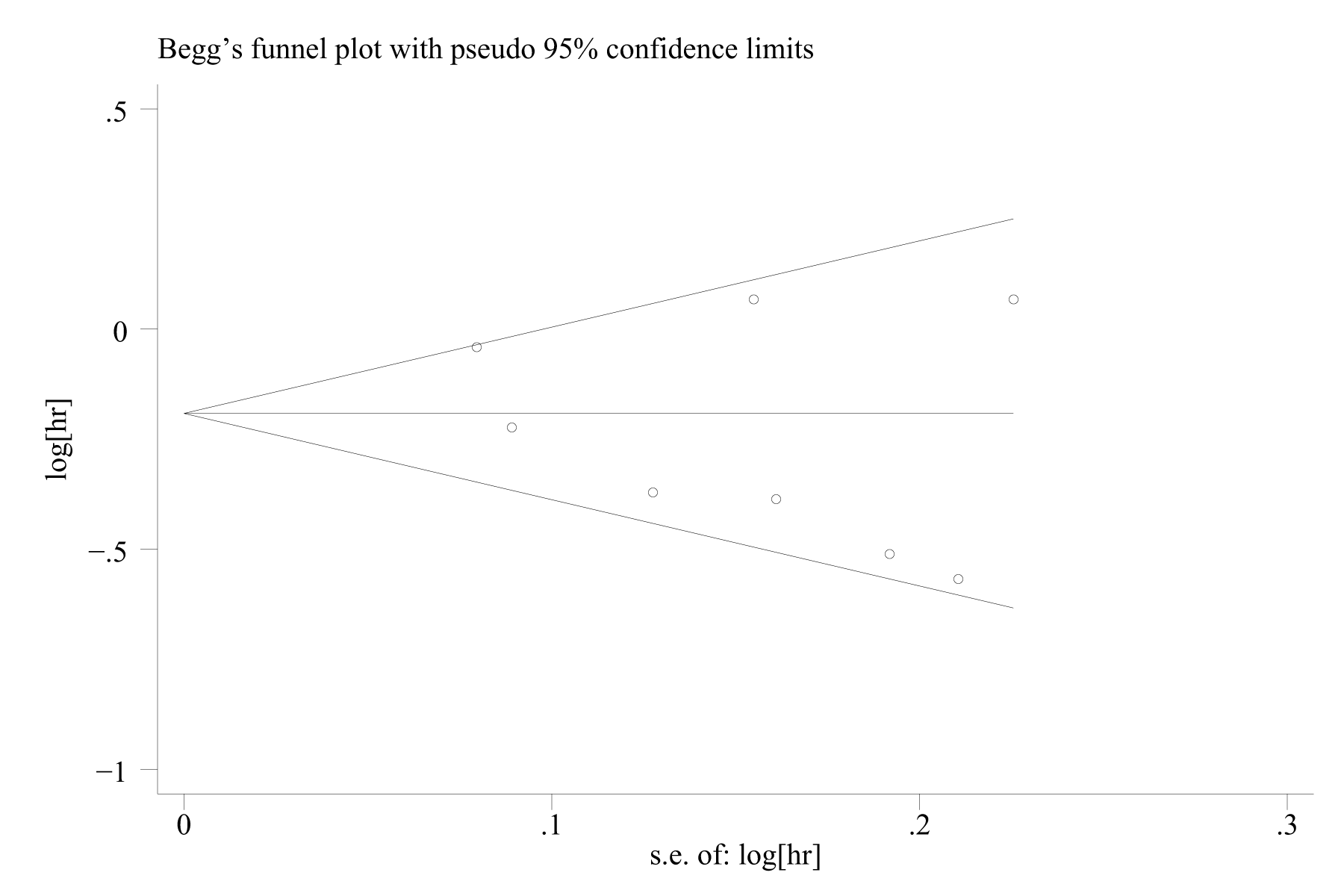

Supplement: Supplementary file 3 — Additional file 3: Supplementary Fig. 3. Begg’s test of the PFS for additional anti-EGFR target agents on RAS/KRAS wild-type patients (p=0.174). [file 12957_2023_3222_MOESM3_ESM.tif]

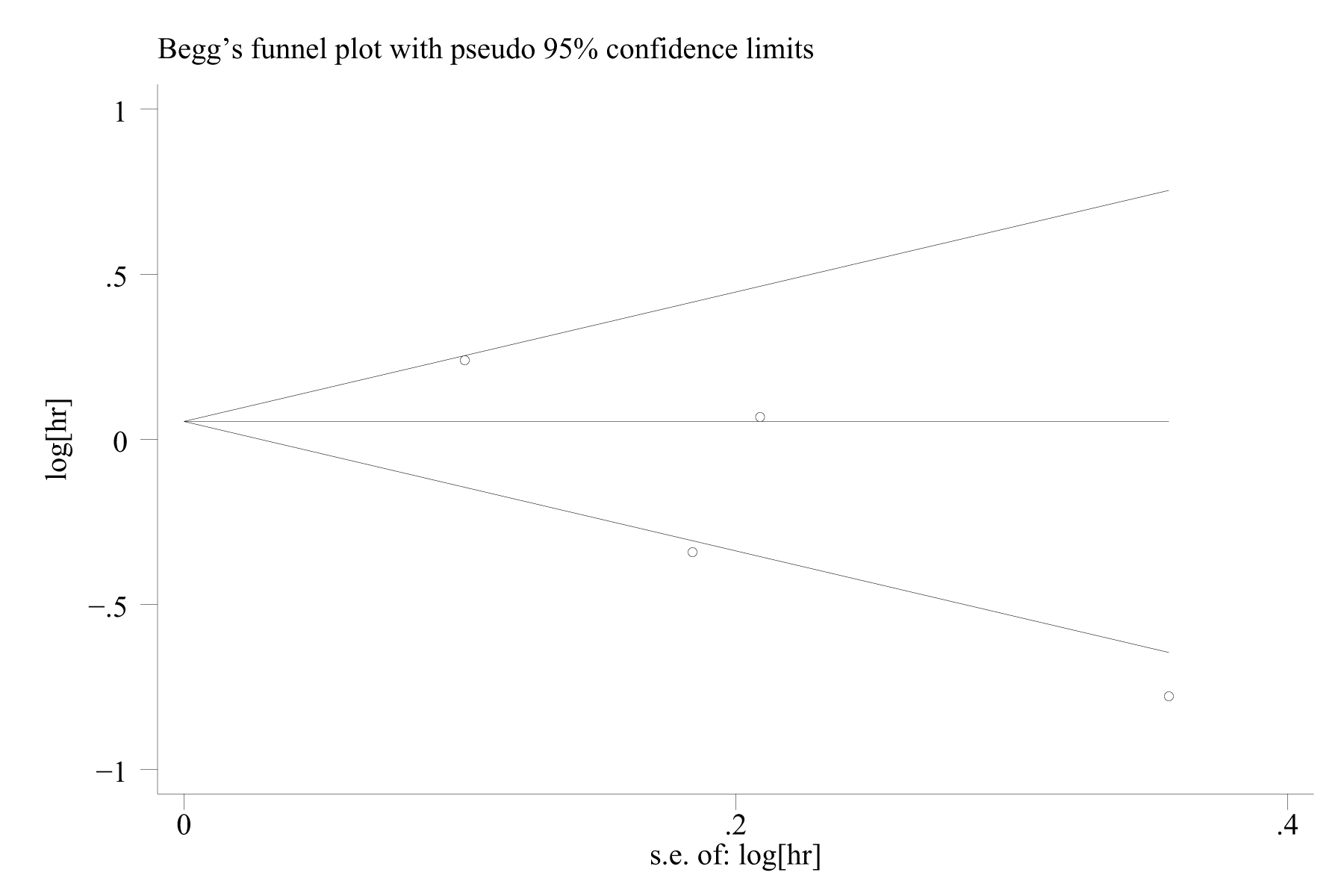

Supplement: Supplementary file 4 — Additional file 4: Supplementary Fig. 4. Begg’s test of the PFS for additional anti-EGFR target agents on KRAS mutant patients (p=0.308). [file 12957_2023_3222_MOESM4_ESM.tif]

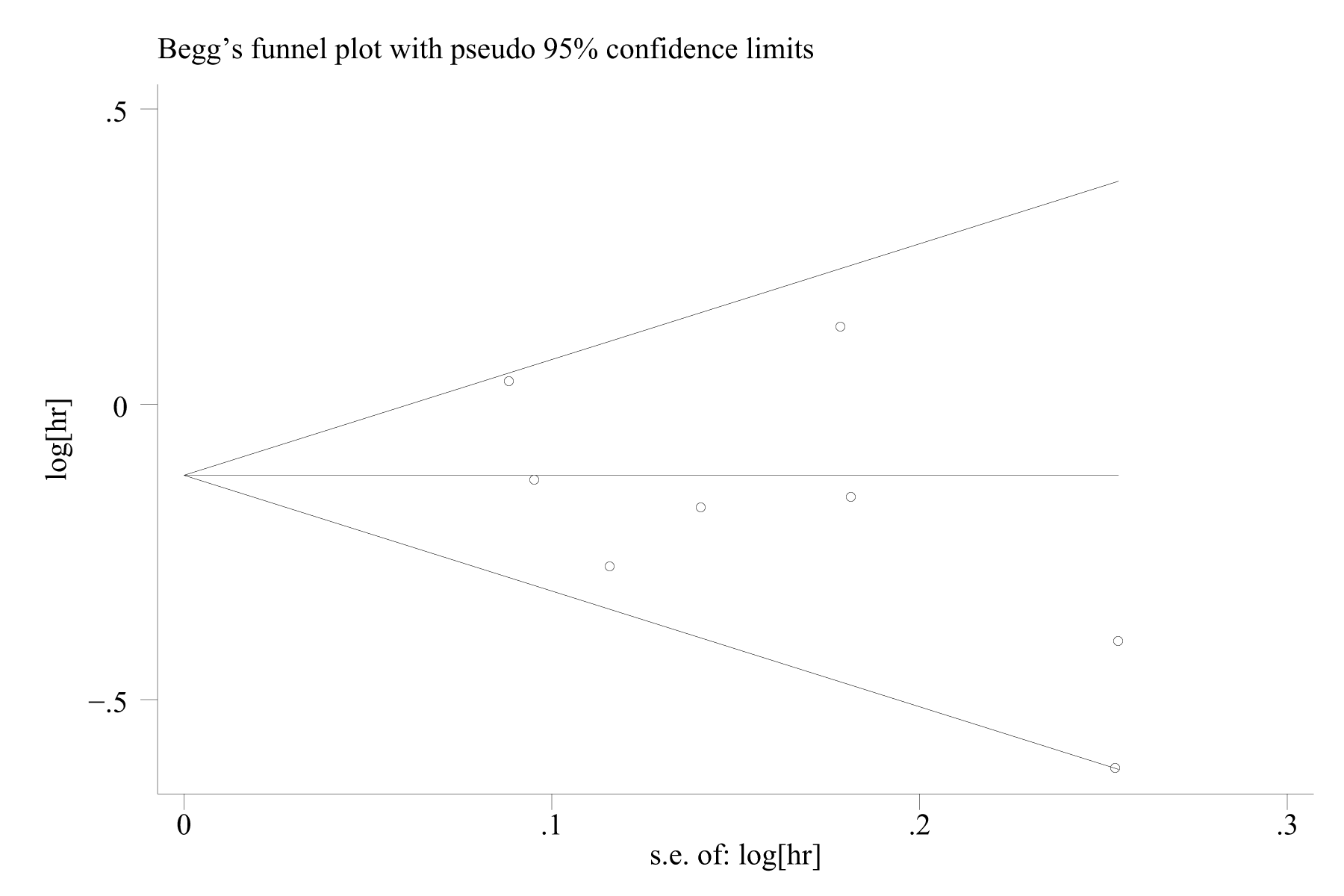

Supplement: Supplementary file 5 — Additional file 5: Supplementary Fig. 5. Begg’s test of the OS for additional anti-EGFR target agents on RAS/KRAS wild-type patients (p=0.174). [file 12957_2023_3222_MOESM5_ESM.tif]

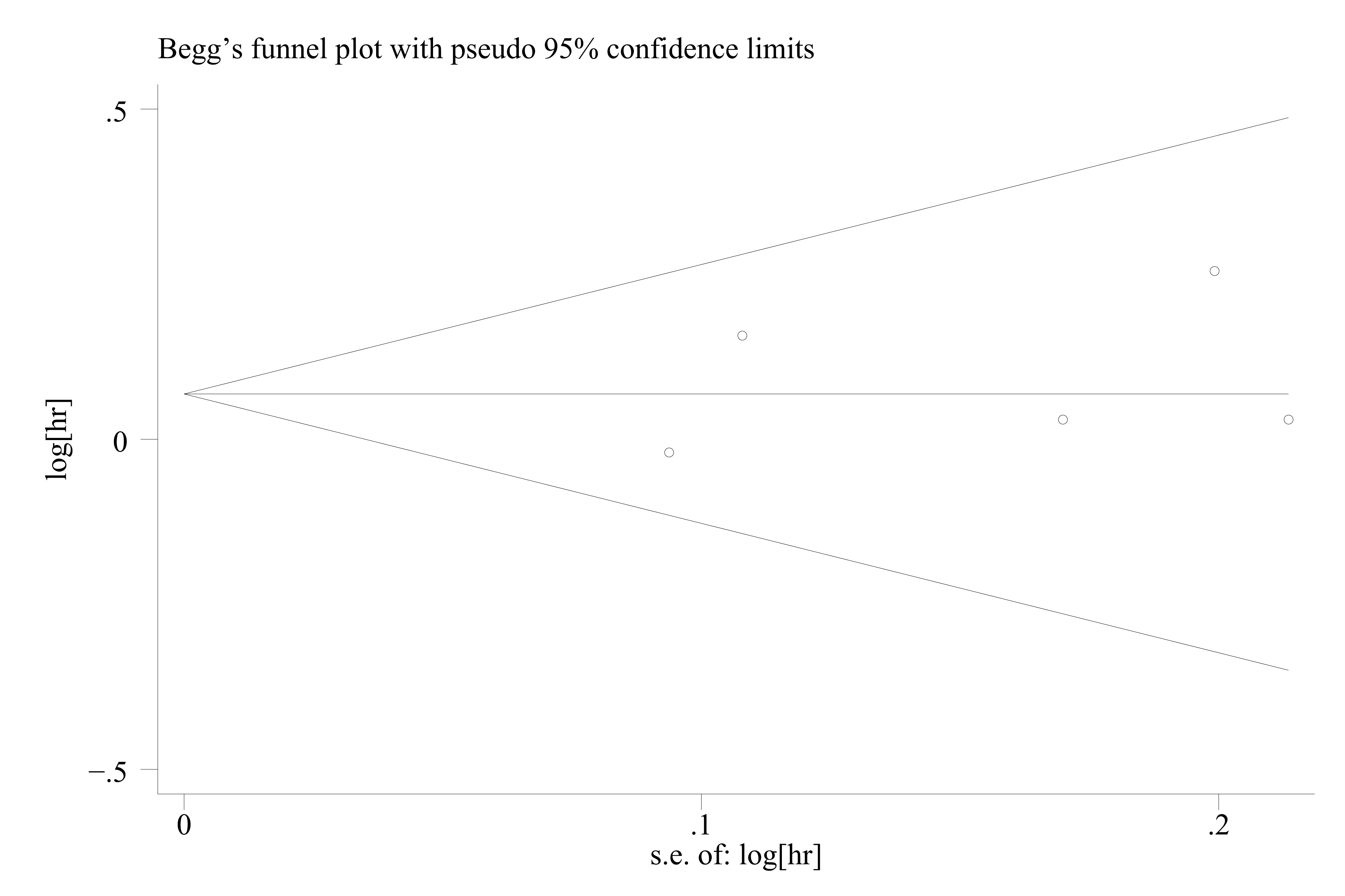

Supplement: Supplementary file 6 — Additional file 6: Supplementary Fig. 6. Begg’s test of the OS for additional anti-EGFR target agents on KRAS mutant patients (p=0.806). [file 12957_2023_3222_MOESM6_ESM.tif]

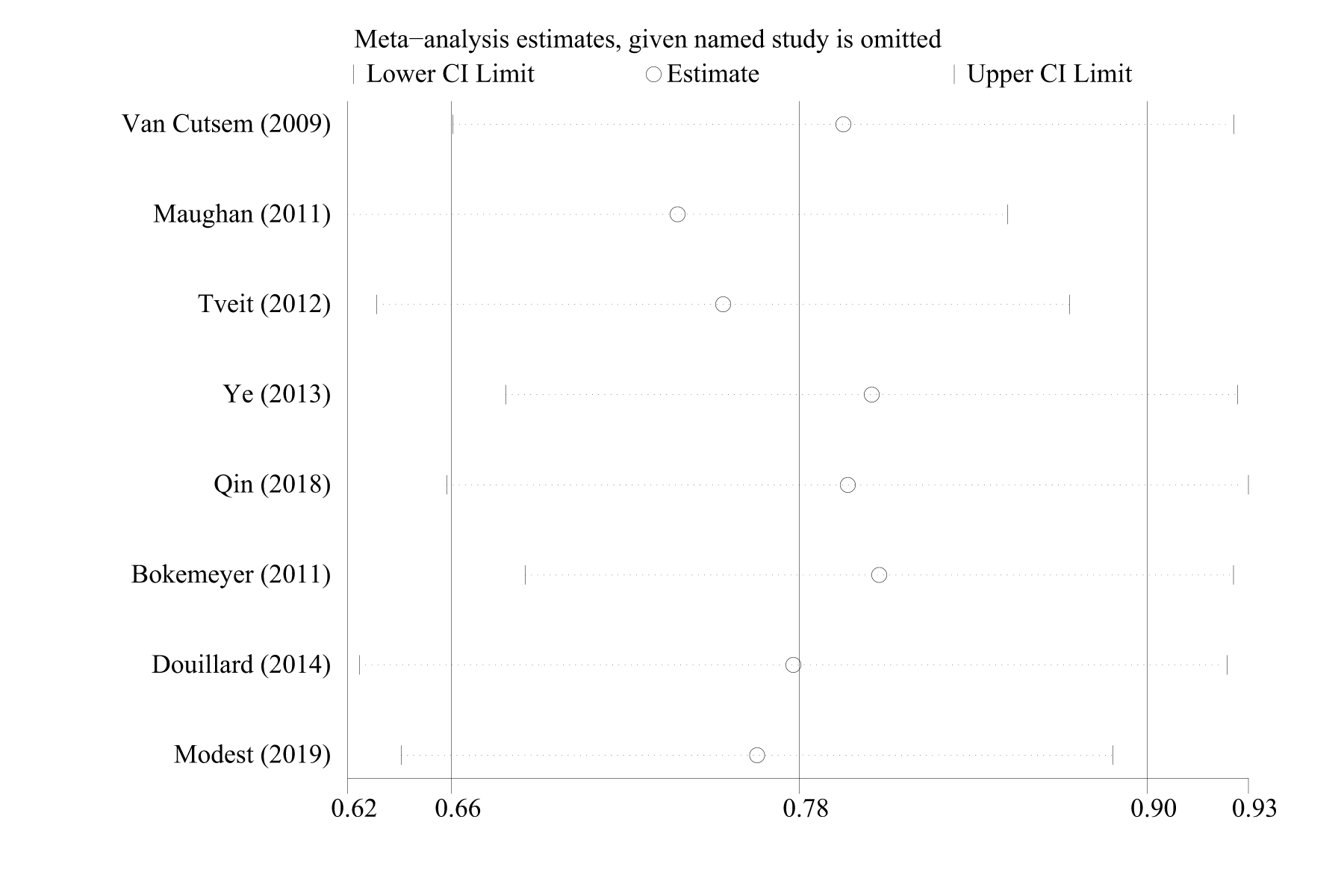

Supplement: Supplementary file 7 — Additional file 7: Supplementary Fig. 7. Sensitivity analysis of the PFS for additional anti-EGFR target agents on RAS/KRAS wild-type patients. [file 12957_2023_3222_MOESM7_ESM.tif]

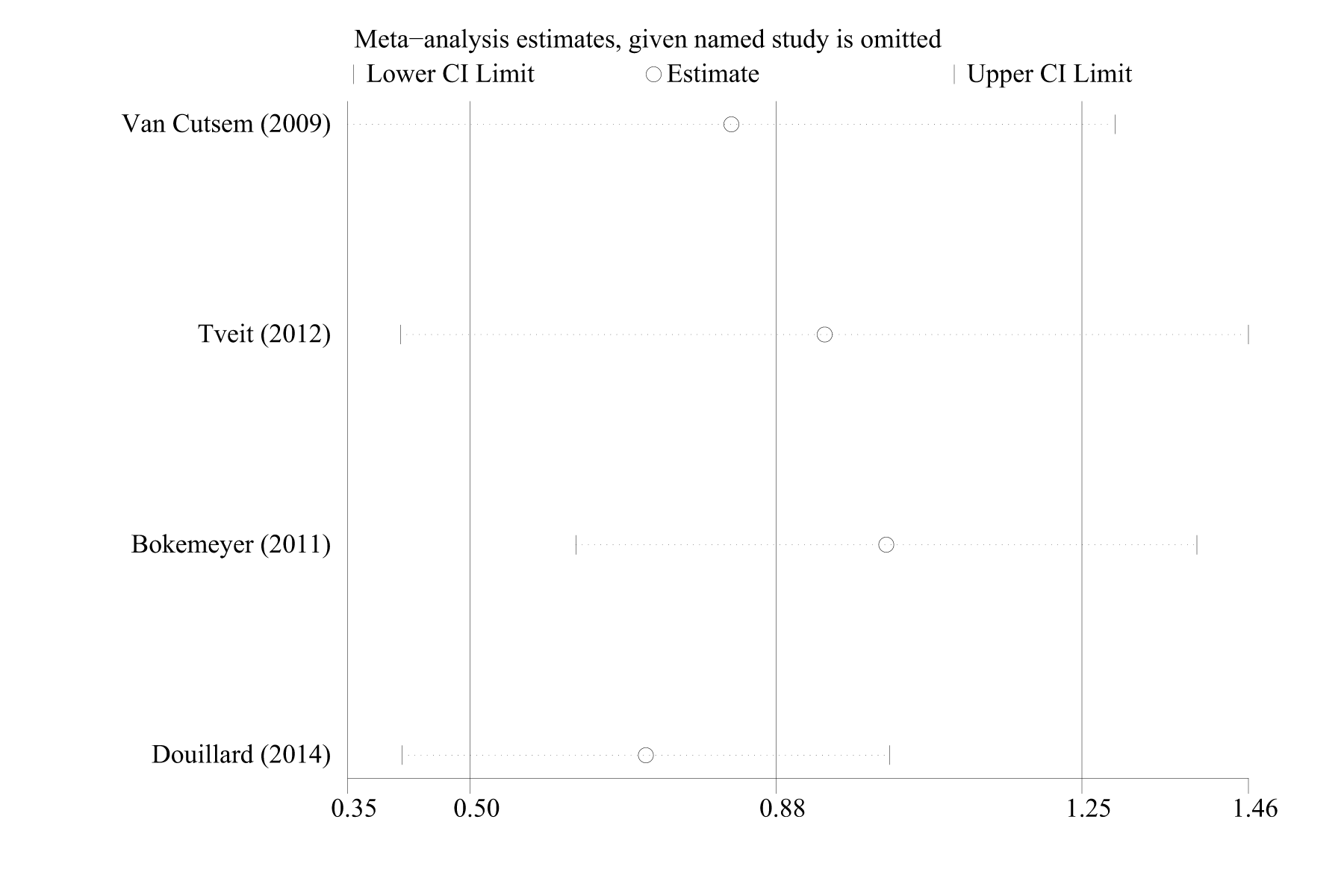

Supplement: Supplementary file 8 — Additional file 8: Supplementary Fig. 8. Sensitivity analysis of the PFS for additional anti-EGFR target agents on KRAS mutant patients. [file 12957_2023_3222_MOESM8_ESM.tif]

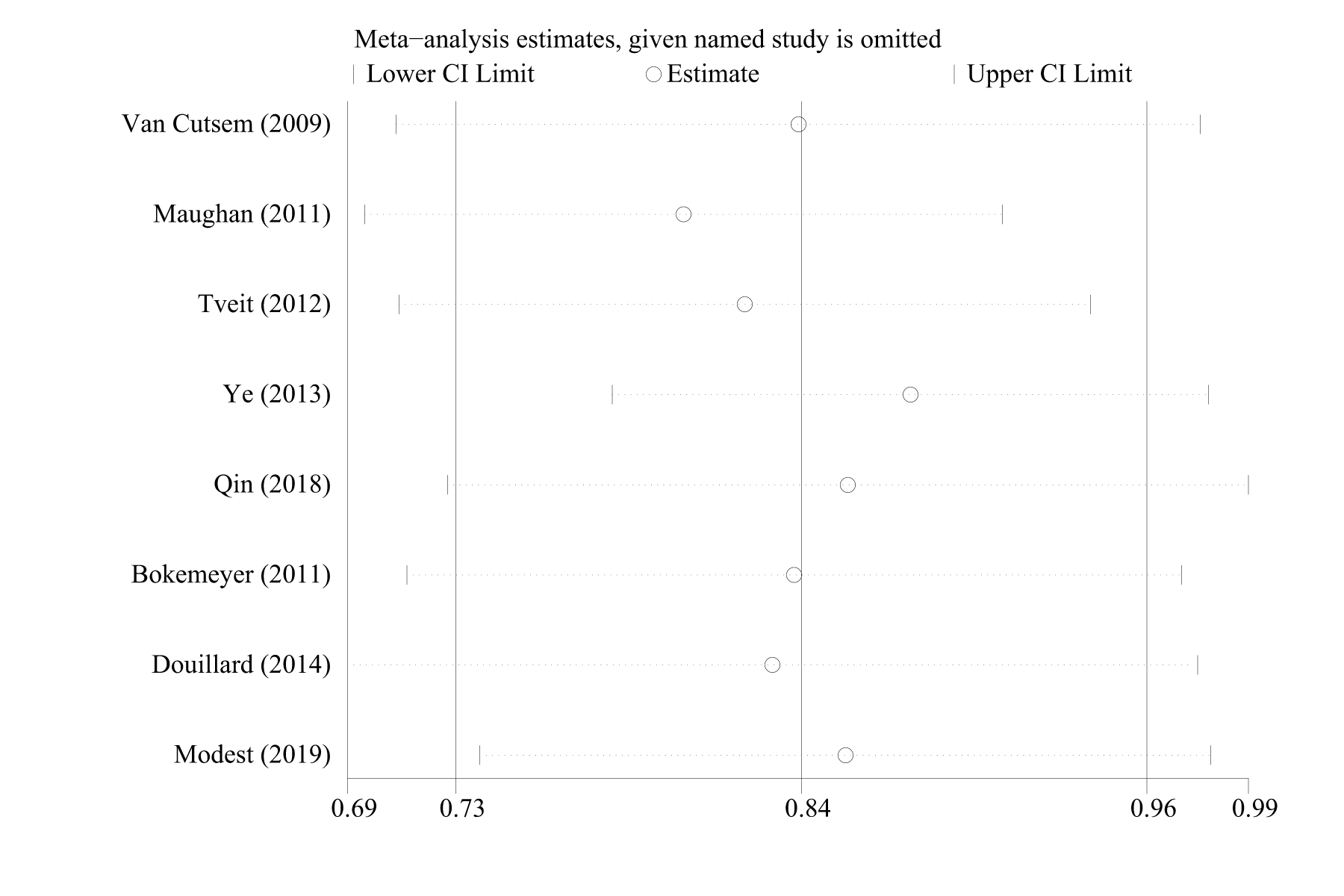

Supplement: Supplementary file 9 — Additional file 9: Supplementary Fig. 9. Sensitivity analysis of the OS for additional anti-EGFR target agents on RAS/KRAS wild-type patients. [file 12957_2023_3222_MOESM9_ESM.tif]

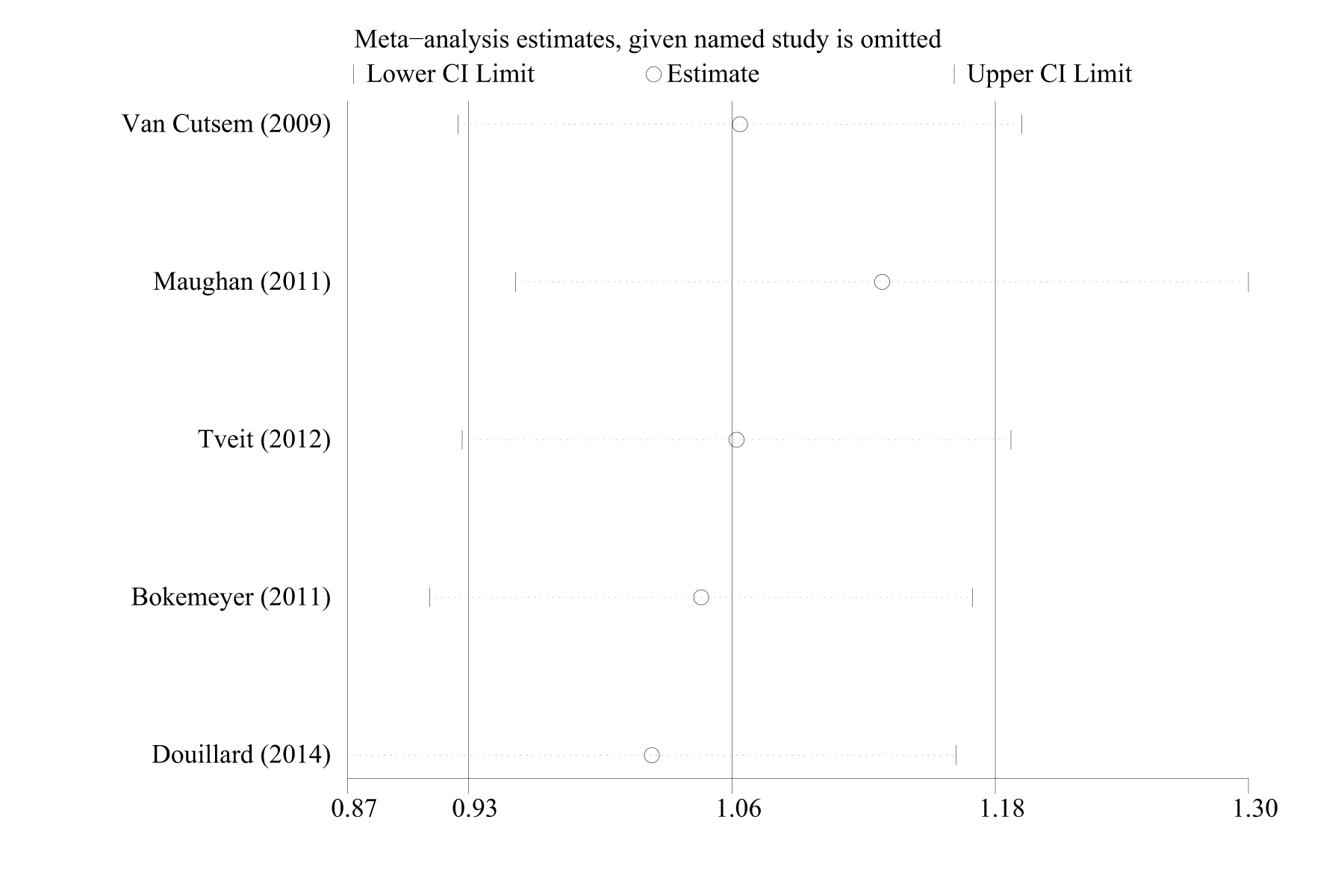

Supplement: Supplementary file 10 — Additional file 10: Supplementary Fig. 10. Sensitivity analysis of the OS for additional anti-EGFR target agents on KRAS mutant patients. [file 12957_2023_3222_MOESM10_ESM.tif]
