# Supplementary material for: The efficacy and safety of anti-EGFR target agents in patients with potentially resectable metastatic colorectal cancer: a meta-analysis of randomized controlled trials
Source: World J Surg Oncol. 2023 Oct 26;21:340. doi: 10.1186/s12957-023-03222-3 (PMC10601219; doi:10.1186/s12957-023-03222-3)
Supplement: Supplementary file 11 — Additional file 11: Supplementary Table 1. Literature search strategy. [file 12957_2023_3222_MOESM11_ESM.docx]

**Supplementary Table 1. Search Strategy.**

**Data searched from PubMed**

| Search Query [All Fields] | Results |
| --- | --- |
| #1 anti-EGFR targeted agents OR epidermal growth factor receptor targeted agents OR panitumumab OR cetuximab | 20,382 |
| #2 colorectal liver metastasis OR metastatic colorectal cancer | 52,638 |
| #3 #1 AND #2 | 3,027 |

Searched on January 10, 2023. Results: 3,027.

**Data searched from Web of Science**

| Search Query [Topic] | Results |
| --- | --- |
| #1 anti-EGFR targeted agents OR epidermal growth factor receptor targeted agents OR panitumumab OR cetuximab | 33,787 |
| #2 colorectal liver metastasis OR metastatic colorectal cancer | 65,016 |
| #3 #1 AND #2 | 6,566 |

Searched on January 10, 2023. Results: 6,766.

**Data searched from** **Embase**

| Search Query [exp] | Results |
| --- | --- |
| #1 anti-EGFR targeted agents OR epidermal growth factor receptor targeted agents OR panitumumab OR cetuximab | 41,967 |
| #2 colorectal liver metastasis OR metastatic colorectal cancer | 55,745 |
| #3 #1 OR #2 | 5,826 |

Searched on January 10, 2023. Results: 5,826.

**Data searched from Cochrane Library**

| Search Query [ti, ab, kw] | Results |
| --- | --- |
| #1 anti-EGFR targeted agents OR epidermal growth factor receptor targeted agents OR panitumumab OR cetuximab | 3,407 |
| #2 colorectal liver metastasis OR metastatic colorectal cancer | 5,460 |
| #3 #1 OR #2 | 1,336 |

Searched on January 10, 2023. Results: 1,336.
