# Supplementary material for: The efficacy and safety of anti-EGFR target agents in patients with potentially resectable metastatic colorectal cancer: a meta-analysis of randomized controlled trials
Source: World J Surg Oncol. 2023 Oct 26;21:340. doi: 10.1186/s12957-023-03222-3 (PMC10601219; doi:10.1186/s12957-023-03222-3)
Supplement: Supplementary file 12 — Additional file 12: upplementary Table 2. Characteristics of all the studies included in the meta-analysis. [file 12957_2023_3222_MOESM12_ESM.doc]

**Supplementary Table 2. Characteristics of all the studies included in the meta-analysis.**

| Author | Year | Recruitment time | Gender (Male/Female) | | Geographic region | | | |
| --- | --- | --- | --- | --- | --- | --- | --- | --- |
|  |  |  | Experiment | Control | Experiment | | Control | |
|  |  |  |  |  | Europe | Rest of the world | Europe | Rest of the world |
| Van Cutsem | 2009 | 2004.7-2005.11 | 713/440 | 636/318 | 975 | 178 | 968 | 157 |
| Bokemeyer | 2011 | 2005.7-2006.3 | 291/268 | 315/257 | 559 | 0 | 572 | 0 |
| Maughan | 2011 | 2005.3-2008.5 | 988/486 | 939/511 | NA | NA | NA | NA |
| Tveit | 2012 | 2005.5-2007,10 | 241/159 | 222/178 | NA | NA | NA | NA |
| Ye | 2013 | 2008.6-2011.12 | 24/46 | 26/42 | NA | NA | NA | NA |
| Douillard | 2014 | 2006.8-2008.2 | 362/189 | 332/218 | 312 | 234 | 306 | 224 |
| Qin | 2018 | 2010-2016 | 127/66 | 139/61 | NA | NA | NA | NA |
| Modest | 2019 | 2011-2016 | 41/22 | 24/9 | NA | NA | NA | NA |

FOLFOX-4, Leucovorin, Fluorouracil, and Oxaliplatin; FOLFOXIRI, flfluorouracil/folinic acid, oxaliplatin, and irinotecan; FLOX, bolus flfluorouracil/folinic acid and oxaliplatin; FOLFOX, 5-fluorouracil, folinic acid and oxaliplatin; NA, not available.
